# Superior Haplotypes for Early Root Vigor Traits in Rice Under Dry Direct Seeded Low Nitrogen Condition Through Genome Wide Association Mapping

**DOI:** 10.3389/fpls.2022.911775

**Published:** 2022-07-08

**Authors:** Annamalai Anandan, Siddharth Panda, S. Sabarinathan, Anthony J. Travis, Gareth J. Norton, Adam H. Price

**Affiliations:** ^1^Crop Improvement Division, Indian Council of Agricultural Research (ICAR)-National Rice Research Institute (NRRI), Cuttack, India; ^2^Indian Council of Agricultural Research (ICAR)-Indian Institute of Seed Science (IISS), Bengaluru, India; ^3^Department of Plant Breeding and Genetics, Odisha University of Agriculture & Technology, Bhubaneswar, India; ^4^School of Biological Sciences, University of Aberdeen, Aberdeen, United Kingdom

**Keywords:** rice, root, root vigor, *aus*, nitrogen, haplotype, genome-wide association study (GWAS)

## Abstract

Water and land resources have been aggressively exploited in the recent decades to meet the growing demands for food. The changing climate has prompted rice scientists and farmers of the tropics and subtropics to adopt the direct seeded rice (DSR) system. DSR system of rice cultivation significantly reduces freshwater consumption and labor requirements, while increasing system productivity, resource use efficiency, and reducing greenhouse gas emissions. Early root vigor is an essential trait required in an ideal DSR system of rice cultivation to ensure a good crop stand, adequate uptake of water, nutrients and compete with weeds. The *aus* subpopulation which is adapted for DSR was evaluated to understand the biology of early root growth under limited nitrogen conditions over two seasons under two-time points (14 and 28 days). The correlation study identified a positive association between shoot dry weight and root dry weight. The genome-wide association study was conducted on root traits of 14 and 28 days with 2 million single-nucleotide polymorphisms (SNPs) using an efficient mixed model. QTLs over a significant threshold of *p* < 0.0001 and a 10% false discovery rate were selected to identify genes involved in root growth related to root architecture and nutrient acquisition from 97 QTLs. Candidate genes under these QTLs were explored. On chromosome 4, around 30 Mbp are two important peptide transporters (*PTR5* and *PTR6*) involved in mobilizing nitrogen in the root during the early vegetative stage. In addition, several P transporters and expansin genes with superior haplotypes are discussed. A novel QTL from 21.12 to 21.46 Mb on chromosome 7 with two linkage disequilibrium (LD) blocks governing root length at 14 days were identified. The QTLs/candidate genes with superior haplotype for early root vigor reported here could be explored further to develop genotypes for DSR conditions.

## Introduction

Rice (*Oryza sativa* L.), is grown in a variety of environments, covering a wide range of latitudes, altitudes, and hydrologies. It feeds more than half of the world’s population, especially in Asia where it serves as the main source of carbohydrates. The consumption is as high as 510.6 million tons (estimated) worldwide in the crop year 2020-2021.^1^ This data illustrate the high demand for the crop and the ongoing intensive production system. Because of its high-water usage (3,000–5,000 liters of water are used to produce 1 kg of grain) and substantial emissions of the greenhouse gas methane, the traditional transplanted technique of rice farming has prompted particular environmental concerns. This system also carries a high labor cost. A recent UN report in 2021 has stressed the need to move toward a more sustainable approach that can address the ongoing faulty agricultural practices and global climate change by smart planning and coordination of sustainable farming practices (FAO, 2021). In order to change the current practice into a more holistic and environmentally friendly approach, it would be apt to shift from transplanted puddled rice (TPR) to other systems that increase water productivity with a minimum yield penalty.

Direct seeded rice (DSR) is one such approach that has the potential to reduce water consumption and labor requirements significantly, while increasing system productivity, resource use efficiency, and checking greenhouse gas emissions (Panda et al., 2021). In this practice of rice cultivation, the growing seedling has early access to above and below-ground resources and the ability to compete with weeds owing to the absence of any transplanting recovery period (Zhang et al., 2017). The early growth traits, such as the early shoot and root growth, seedling establishment, and root proliferation, which are collectively termed as early seedling vigor, are of utmost importance while developing new varieties for the DSR system (Mahender et al., 2015; Anandan et al., 2016). Surprisingly, the entity that often gets overlooked while studying vigor is the root and its manifestations. A clear understanding of the root system, its development, and ideal requirements for its growth are fundamental while working with the DSR system of rice. This would not only facilitate better seedling establishment but also aid in the proper uptake of nutrients and moisture, which is limited under DSR conditions (Anandan et al., 2016). Nutrient application in the form of synthetic fertilizers has become indiscriminate in the recent past degrading soil health and at the same time increasing expenses. The high-yielding modern-day cultivars are adapted to such high levels of fertilizer inputs but the nutrient use efficiency is much less than 50% of the total application. For instance, nitrogen recovery efficiency in rice remains below 50% of the total fertilizer applied to the crop (Chivenge et al., 2021). Additionally, it has also been found that the application of nitrogenous fertilizers as the basal dose can highly contribute toward weed growth instead of catering to the seedling establishment in the DSR system of cultivation. The new varieties bred for the DSR system should be able to survive the initial seedling stage with a lower level of nitrogen application. This can be facilitated by increasing the nitrogen uptake aided by respective genes, transcription factors, and transporter molecules. Therefore, the mining of key genes for superior haplotypes or genomic marker loci that control the early root vigor facilitating higher nutrient uptake under low nitrogen conditions to reveal the underlying molecular mechanisms can be a promising and important objective in rice breeding.

The broad adaptation of rice as a species, both in DSR and TPR, is associated with large genetic and phenotypic diversity (McNally et al., 2009). A population constructed with accessions from an environmentally defined genetically diverse setting with the trait of interest under strong genetic control can be studied for this purpose. *Aus*, a diverse group that evolved from the annuals *Oryza nivara* found in Bangladesh, Northern Myanmar, and NE India (Kim et al., 2016), is cultivated mainly under rainfed conditions and represents an untapped source of genetic variations. They are phenotypically diverse, harbor a number of abiotic stress-resistance-related genes, and are photoperiod insensitive (Travis et al., 2015). Studying such a population can help us explore and unravel genes for ideal root traits contributing toward early root vigor in DSR conditions.

Root architecture requirements at the seedling stage can be defined from the perspective of seedling establishment and nutrient uptake. The seedling stage would require root growth enhancers that facilitate early seedling establishment. This should be accompanied by a higher number of crown roots and surface roots to acquire most of the immobile nutrients (P, Fe, and Zn) from the surface soil. For instance, phosphorus (P) is an element highly required for root growth; plants start transporting P from the second day of germination (Julia et al., 2018) and, in a hydroponic experiment it was found that seedlings, require at least 6 ppm during the first 2 weeks of sowing (Anandan et al., 2021). Genes/QTLs, such as *qSOR1, PhTs, AMTs, NRTs, qEVV*, *Pstol1*, etc., would help in the initial establishment of the seedling (Panda et al., 2021) and with supplementary assistance from root growth genes like expansins (Yu et al., 2011), *RLCK*s (Ou et al., 2021), *HDAC* (Chung et al., 2009), *GLPT* (Xu et al., 2019), etc. Several reports have shed light on the genetic aspects of early shoot vigor (Mahender et al., 2015; Rebolledo et al., 2015; Sandhu et al., 2015; Anandan et al., 2016; Lee et al., 2017) under DSR conditions. In contrast, early root vigor traits (studied at 14 and 28 days) as a function of the seasonal variability have not been reported earlier, so the results from this study can open up new avenues to explore and exploit for DSR varietal development.

In this perspective, we have used a complementary and powerful tool for connecting the genotype–phenotype map, genome-wide association studies (GWASs), for the discovery of new markers to be used in marker-assisted selection (MAS). Earlier, studies exploiting GWAS have mainly reported QTLs linked to shoot traits influencing seedling vigor. Thus, our objective was to dissect root vigor traits using high-density single-nucleotide polymorphisms (SNPs) between QTLs governing early root and shoot traits and their candidate genes under limited nitrogen conditions in DSR. These SNPs can aid in the identification of superior haplotypes that can be utilized in haplotype-assisted breeding for DSR conditions.

## Materials and Methods

### Plant Material

A total of 298 representatives from the Bengal and Assam Aus Panel (BAAP) developed by Norton et al. (2018) were used to study the root vigor in DSR under low nitrogen soil conditions. This population had 298 accessions, with mostly *aus* landraces (Travis et al., 2015; Norton et al., 2018), 19 of the OryzaSNP panel (McNally et al., 2009) plus some released varieties and breeding lines from Bangladesh. A total of 278 genotypes of the BAAP have been used in this study (Supplementary Table 1). The ∼2 million (2,053,863) SNP dataset, generated using skim sequencing at ∼4 × depth, from this population, is available under the project called ‘‘BAAP’’ in the SNP-Seek database^2^ and on the Harvard DataVerse as a data-set “Genome-Wide Association mapping of grain and straw biomass traits in the rice BAAP” [also available under the project called “BAAP” in the SNP-Seek database (see text footnote 2)] have been utilized in the present study to identify the underlying QTLs and construct haplotypes governing root vigor traits for GWASs.

### Field Screening

The mapping panel of *aus* population was screened under low N (low soil N levels) at ICAR-National Rice Research Institute, Cuttack (20°27′09″N, 85°55′57″E, 26 m.a.s.l.) during two consecutive seasons of 2018 Kharif (wet season) and 2019 Rabi (dry season) in the upland situation, i.e., non-saturated aerobic condition with supplemental surface irrigation. The sandy clay loam soil of our experimental plot recorded 0.53% organic carbon, 220 kg/ha of N, 26.3 kg/ha of P, 164.05 K kg/ha, 51.6% sand, 18% silt, and 30.4% clay with a bulk density of 1.41 g/cm^3^. To assess the early root growth parameters under limited nutrient DSR conditions, N fertilizer was not applied for the entire experimental period of 28 days, while, recommended 100% P (40 kg) and 50% K (20 kg) fertilizer was applied as basal. The subsurface soil compaction was measured using a penetrometer, the resistance ranged between 11 Kpa (15 mm) and 2,148 Kpa (600 mm). In the 2018 wet season, on average the plants received 33.4°C during the day, 25.5°C at night, and sunshine for 3.2 h per day with total rainfall of 190 mm. In the 2019 dry season, plants received an average temperature of 28.15°C during the day, 12.86°C at night, and sunshine for 4.54 h per day with nil rainfall days during the observation period. Four to five seeds of each genotype were sown per hill manually in 3 rows of 1.5 m long, with a spacing of 20 cm between rows and 15 cm between plants in two replicates by following a randomized block experimental design. Weed growth was checked by spraying the pre-emergence herbicide Pendimethalin (at the recommended dose) within 24 h of irrigation. Further hand weeding was done on 15 days after sowing (DAS) to keep the field weed free.

### Sample Collection and Traits Recorded Related to Early Root Vigor

Different vegetative traits concerning root growth were undertaken to assess root vigor (RV) on 14 and 28 DAS. Six rice plants in the middle row from each genotype were selected to study different root and shoot traits. For this, the border plants of the middle rows were excluded, and three consecutive hills were harvested each for 14 and 28 DAS leaving one hill in between the two samples. To have maximum roots during the excavation of plants for observation, the experimental plot was flooded on the day before uprooting. The uprooted seedlings from the field were carefully washed in running tap water to remove soil for measuring root-related parameters such as root length (cm), number of crown roots, and root dry weight (g) on both 14 and 28 DAS. The average root length was measured from the crown of the root to the tip of the root. Shoot weight was also recorded to study the relation between root trait manifestation and shoot biomass accumulation. To observe the dry weight of the shoot and root, the samples were dried in an air-forced oven at 60°C for 5–6 days until the samples were completely dried. These observations were recorded for two seasons as mentioned before.

### Statistical Analysis

Box plot was deployed to derive the various initial descriptive statistics that included mean, SD, minimum and maximum values, coefficient of variation (%), skewness, kurtosis, and distribution pattern. Absolute growth rate (AGR), crop growth rate (CGR), and relative growth rate (RGR) were calculated for the root traits.

Absolute growth rate defines the rate in change of root length and was calculated as follows:


$$\text{AGR}=\left({\text{L}}_{2}-{\text{L}}_{1}\right)/\left({\text{t}}_{2}-{\text{t}}_{1}\right)\,\,\left(\mathrm{in}\,\mathrm{cm}\,{\mathrm{day}}^{-1}\right)$$

where L_1_ and L_2_ are the root length at times t_1 (14th  day)_ and t_2 (28th  day)_, respectively.

CGR measures the root dry weight of a particular ground area at a regular interval of time divided by land area and represented as g m^–2^ day^–1^.


$$\text{CGR}=\left({\text{W}}_{2}-{\text{W}}_{1}\right)/\text{P}\,\left({\text{t}}_{2}-{\text{t}}_{1}\right)\,\,\left(\text{g}/{\text{m}}^{2}/\text{day}\right)$$

where W_1_ and W_2_ are the root dry weights at times t_1 (14th  day)_ and t_2 (28th  day)_, respectively, P = spacing (m^2^).

RGR defines the rate of change in logarithmic root dry weight as follows in mg g^–1^ day^–1^.


$$\text{RGR}=\left({\text{log}}_{\text{e}}\,{\text{W}}_{2}-{\text{log}}_{\text{e}}\,{\text{W}}_{1}\right)/\left({\text{t}}_{2}-{\text{t}}_{1}\right)\,\,\left(\text{mg}/\text{g}/\text{day}\right)$$

where W_1_ and W_2_ are the root dry weights at times t_1 (14th  day)_ and t_2 (28th  day)_, respectively.

The variability in traits related to root vigor was described using a PCA. PCA analysis was carried out using the FactoMine R package (Lê et al., 2008) in R 3.6.4 (R Core Team, 2017). Euclidean distance between two genotypes was estimated by analyzing PCA in the multivariate space and identification of genotypes having superior root vigor traits under DSR conditions. To understand the relationship between different traits, the general correlation among all the shoot and root traits was analyzed using the corrplot functions from the corrplot package (Wei and Simko, 2021) in R 3.6.4.

### Genome-Wide Association Mapping

The BAAP population was subjected to GWA mapping using the PIQUE pipeline in order to preprocess the genotype and phenotype data (Norton et al., 2018) for subsequent EMMAX analyses on each phenotype in parallel. The minor allele frequency (MAF) was set at 0.05 and SNPs below it were filtered out, the maximum missing percentage per SNP was set at 5%. The GWA mapping between each SNP and trait values for each genotype of the population was done using an efficient mixed effect model (EMMA) simultaneously accounting for the population structure and cryptic kinship with a significance threshold of *p* < 0.0001 to identify significant SNPs. Benjamini–Hochberg adjusted probabilities were calculated using the false discovery rate (FDR) of detected associations. The significance threshold of FDR was maintained at 10% to identify putative SNP associations (McCouch et al., 2016) and were represented in the Manhattan plots. After GWA, SNPs with −log_10_(P) < 4 were examined to group the SNPs into QTLs. SNPs that were closer than the genome average linkage disequilibrium (LD) decay value (of 243 Kbp) were assumed to be of the same locus.

Based on the decay of LD, a sliding window was defined to bin together the significant SNPs into peaks, using PLINK (Purcell et al., 2007) with parameters “-clump-p1 0.0001 -clump-p2 0.0001 -clump-r2 0.3 -clump-kb 243.” SNPs that were closer than the BAAP genome average LD decay value (of 243 Kbp) were assumed to be of the same locus. This procedure was done for each trait separately. Pairwise *r*^2^-values were calculated for every SNP with *p* < 0.0001 and the surrounding SNPs were within the range of 243 kb and also had a *p* < 0.0001. Any two such SNPs that met these criteria along with an *r*^2^ ≥ 0.3 were clumped into bins. Binning was performed for each experiment and trait studied. SNPs that did not meet the above criteria were considered singleton. The QTLs were reported only when they harbored at least one significant SNP (*p* > 0.0001) with a 10% FDR.

### Identification of Candidate Genes and Haplotype Analysis

For each QTL, the LD decay value of 243 kbp was considered around the peak SNP and all the genes present in this stretch were annotated using the Rice Genome Annotation Project (RGAP)^3^, release 7. The genes with annotations such as “(retro) transposon” and “hypothetical” were excluded from the identification of candidate gene analysis. To understand the gene function and ontology, the RGAP website was used. Additionally, all the available research reports and other online resources were searched exhaustively for any information regarding the genes in the LD block. Any gene that currently has a function related to root vigor including growth, development, or regulation of root metabolic activities was taken into account. Then, the level of expression of these shortlisted genes in the roots was checked from the Rice Expression Profile Database (*RiceXpro*) database. The *RiceXpro* repository provides tissue-specific profiles of expression for every gene in the reference cultivar Nipponbare. Only those genes that were expressed in the roots and had a definitive role in the traits related to root vigor and also present in the same LD block as that of the peak SNP were selected as candidate genes. QTLs were identified across traits and/or seasons to be associated with various root traits significantly and the LD heatmap surrounding the peaks of these QTLs was constructed using the “LDheatmap,” an R package that uses squared Pearson’s correlation coefficient (*r*^2^). The SNPs that were significantly associated with the root traits studied were extracted using PLINK. The non-synonymous and synonymous SNPs within the exon regions of the candidate genes were identified on the Nipponbare reference genome from the *snp-seek.irri.org* database. These SNPs in the exon regions were used to group the population into haplotypes and the phenotypic mean performance of each haplotype was determined.

## Results

### Phenotypic Variations and Correlation Among the Early Root Traits Under Low Nitrogen Direct Seeded Rice Conditions

**FIGURE 1:**
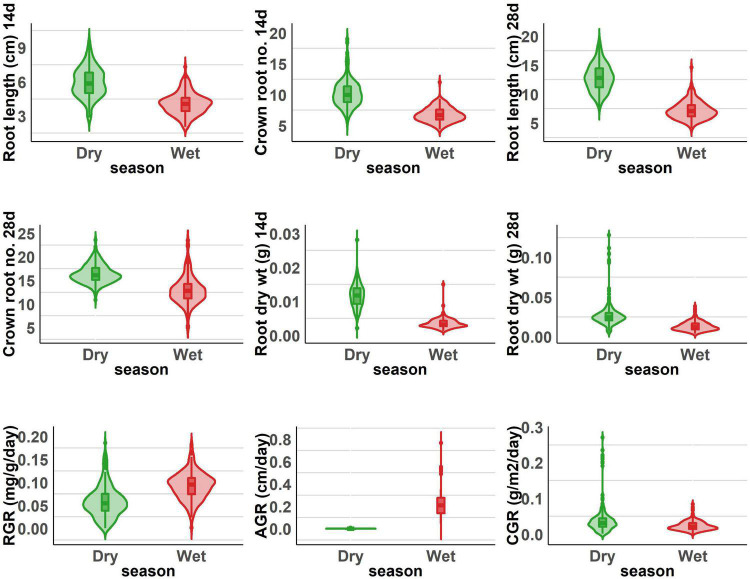
Root length, dry weight, crown root number, and root vigor index of dry and wet seasons under DSR conditions.

**FIGURE 2:**
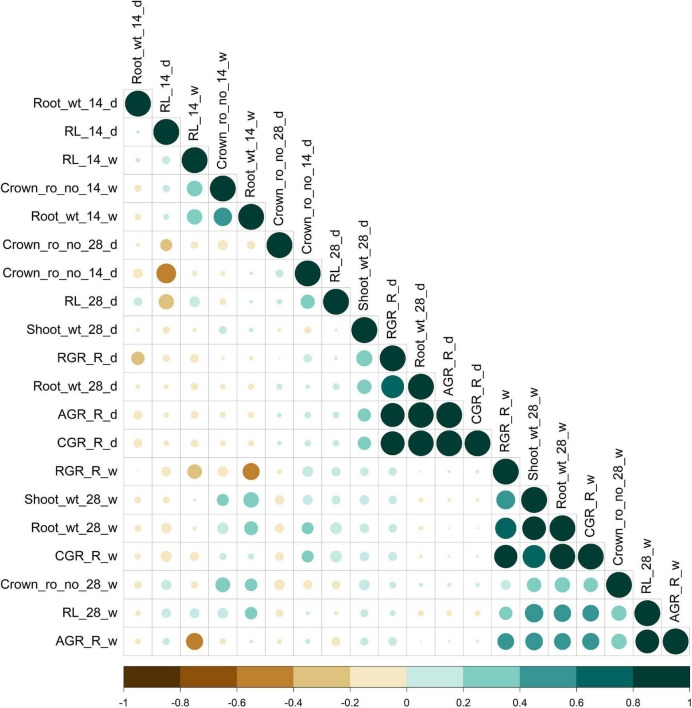
Pearson correlation for root length, dry weight, crown root number, and root vigor index of dry (d) and wet (w) season under DSR condition. Color (green, positive correlation; brown, negative correlation) intensity and the size of the circle are proportional to the correlation coefficient.

The population displayed a great deal of variation, significant for all the traits under consideration in this study. A significant difference was observed in the response of the BAAP genotypes between the two seasons; dry and wet seasons (Figure 1 and Supplementary Table 2). Only one trait; crown root number at 28 days showed significant genotype × season treatment interactions (Table 1). All of the studied traits exhibited higher mean values in the dry season as compared to the wet season (except for AGR and RGR). The mean root length at 14 and 28 days was 5.97 and 12.83 cm, respectively, as opposed to 3.97 and 7.16 cm, respectively, in the dry season. The crown root number at 14 days was as high as 10.2 in the dry season. The AGR was high in the wet season with a value of 0.277 g day^–1^ while in the dry season it was 0.001 g day^–1^. The mean shoot dry weight at 28 days however recorded the same value of 0.63 g both in dry and wet seasons. Correlation studies done in the population revealed a highly positive correlation between the root dry weight (28 days) with RGR (0.78), and CGR (0.97) in the dry season (Figure 2 and Supplementary Table 3). In the wet season, root dry weight at 28 days displayed a positive correlation at a high degree with RGR (0.68) and CGR (0.97) while root length at 28 days had a positive correlation with AGR (0.84). It is noteworthy to mention that shoot weight and root weight at 28 days showed a high positive correlation (0.84).

**TABLE 1:** The percentage contribution of genotypes, season and genotype × season interactions for the root traits observed under direct-seeded condition.

Traits	Genotype (G)	Season (S)	G × S	G × S
Root length (14 days)	20.17**	15.08**	NS	15.13
Crown root no. (14 days)	38.72**	12.28**	NS	10.06
Root dry weight (14 days)	66.67**	8.33**	NS	8.33
Root length (28 days)	60.30**	9.12**	NS	8.02
Crown root no. (28 days)	29.27**	15.30**	20.94*	20.94*
Root dry weight (28 days)	18.46**	20.51**	NS	20.00
Relative growth rate	13.57**	24.37**	NS	20.69
Absolute growth rate	35.17**	11.09**	NS	13.02
Crop growth rate	5.92**	24.42**	NS	23.47

*p < 0.05, **p < 0.01.

### Principal Component Analysis Among the Early Root Traits Under Low Nitrogen in Direct Seeded Rice Conditions

**FIGURE 3:**
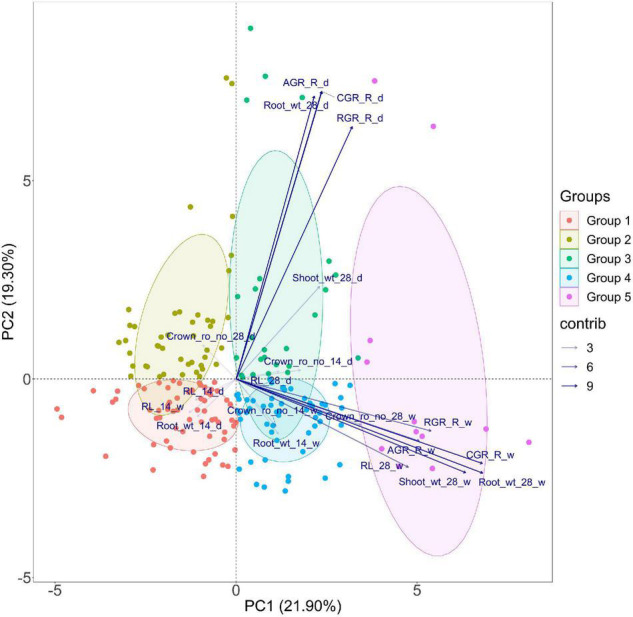
PCA for root length, dry weight, crown root number, and root vigor index of dry (d) and wet (w) season under DSR conditions explained by two axes. Together, the two PC axes explained 41.2% of the total variance. The transparency of the vector indicates the contribution to the variance in the dataset, ranging from 3 (lightest) to 9% (darkest). The direction and length of the vector represent the trait contribution to the first two components of the PCA. Genotypes are grouped into five based on their expression pattern of root traits measured under DSR conditions. Group 1 (solid red tone) and Group 2 (solid algae tone) share poor root vigor parameters, Group 3 (solid green tone) performed well during the dry season, Group 4 (solid cyan tone), and Group 5 (solid purple tone) share high values for root vigor traits during the dry season at 28 days and wet season.

Before venturing into the GWAS, a PCA was performed to visualize the grouping pattern and highlight the traits that contribute the most to the total variance observed in the population subjected to low nitrogen stress under direct-seeded conditions. The data from the two seasons, *viz.*, dry and wet seasons, at 14 and 28 DAS were subjected to PCA, and it highlighted six principal components with eigen values of more than 1. However, only three components explained variations accounting for more than 10% individually. PC1, PC2, and PC3 explained 21.9, 19.3, and 11.81% variations, respectively. Of all the traits, root dry weight (28 days) and CGR had one of the highest coefficients in both dimensions 1 and 2. PC1 showed greater expression for traits like crown root number, root length, root dry weight at 28 days, RGR, AGR, and CGR in the wet season. PC2 showed positive values for the traits root dry weight at 28 days, RGR, AGR, and CGR in the dry season (Figure 3 and Supplementary Table 4). Of all the traits studied, crown root number, root dry weight, and root length at 28 days were positively correlated in both seasons. Additionally, shoot dry weight at 28 days positively correlated with root dry weight at 28 days in both seasons.

### Subpopulation Statistics

#### *Aus* Subpopulation Analysis

**FIGURE 4:**
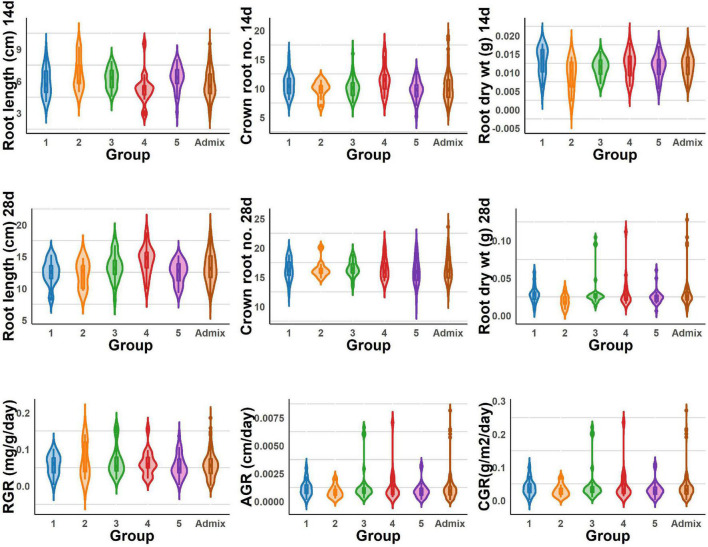
Violin graph represents the range of root length, dry weight, crown root number of 14 and 28 days, and root vigor index variations under DSR conditions of different BAAP groups in the dry season.

**FIGURE 5:**
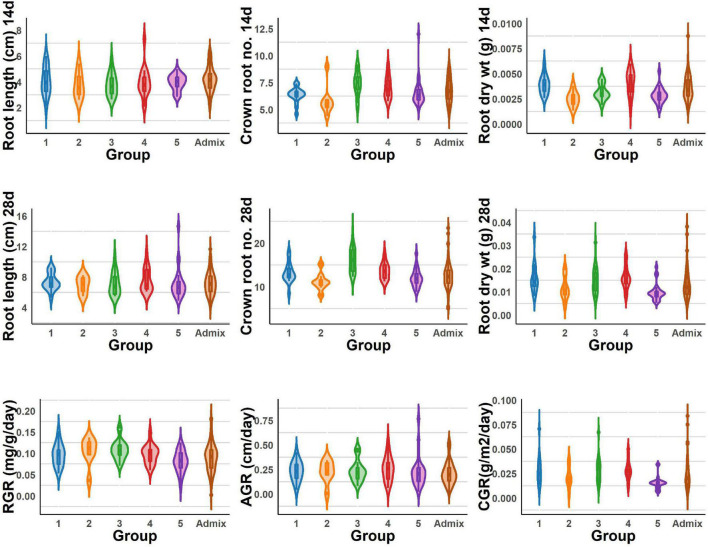
Violin graph represents the range root length, dry weight, crown root number of 14 and 28 days, and root vigor index variations under DSR conditions of different BAAP groups in the wet season.

The BAAP population manifests in five groups based on structure analysis of the 5.2 million SNPs (Norton et al., 2018), and one-way ANOVA revealed significant variations among these five groups for all the traits studied for early root vigor (Figures 4, 5). The genotypes in Group 4 performed above average for all the traits across seasons except for root length (14 days) and root RGR in the dry season. In contrast, Group 2 exhibited lower mean values compared with other counterparts except for root length (14 days) and root RGR in the dry season. In the wet season, Groups 4 and 5 exhibited superiority for most of the traits. Of all the groups, the admixtures performed the better for most of the characters across the seasons.

### Genome-Wide Association Mapping of Root Vigor-Related Traits

**FIGURE 6:**
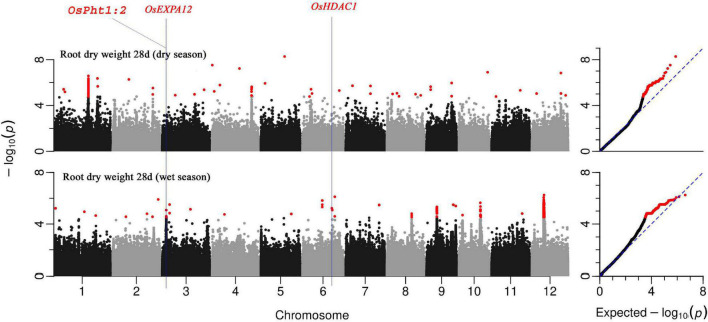
Manhattan plots from GWA mapping of root dry weight at 28 days under DSR condition of BAAP population in wet and dry seasons. Benjamini–Hochberg adjusted probabilities > 0.1 are highlighted in the red dot. The diagonal blue line shown on QQ Plots represents a 1:1 agreement between expected probability.

Genome-wide association mapping conducted on the BAAP population highlighted significant genomic loci, associated QTLs, and genes that regulate various root vigor-related traits at the early seedling stage of rice in DSR conditions under low nitrogen soil status. A total of 97 QTLs associated with at least one of the studied root traits were identified across seasons (Supplementary Table 5). Few of these QTLs were detected across traits while others were unique. A list of 275 unique putative genes all related to the traits under study was identified in these 97 QTLs of which nine genes were repetitive across different traits (Supplementary Table 6). These genes were all selected based on earlier reports in https://shigen.nig.ac.jp/rice/oryzabase/database and their level of expression in the *RiceXpro* database. Of these, 134 genes (Figure 6 and Supplementary Figures 1, 2) were root morphology regulating traits, 48 genes were having auxin-related functions, 13 were amino acid-related genes, and 3 were aquaporin proteins. The rest of the genes were associated with nutrient uptake and utilization; 9 were mycorrhizae-associated genes, 26 were related to nitrogen, 30 were related to phosphorus, 4 with potassium, 8 with iron, 2 with sulfur, and 1 with zinc. Of all these QTLs, six QTLs that were significantly associated with early root vigor traits with higher gene expression (observed in the *RiceXpro* database; the corresponding genes were found to be highly expressed in roots at the vegetative stage) in the roots were selected for haplotype analysis. These QTLs were distributed across chromosomes, one for crown root number in chromosome 6, two for root dry weight in chromosome 3, two for root length in chromosome 7, and one for AGR in chromosome 4.

### Haplotype Analyses

**FIGURE 7:**
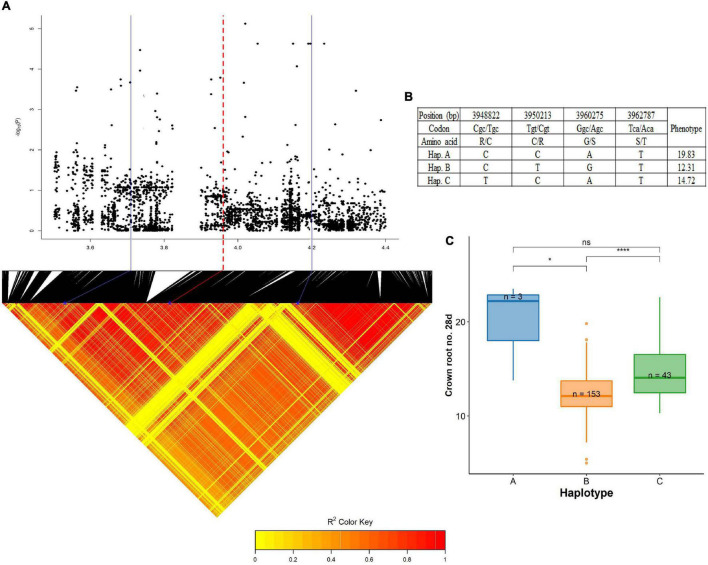
Significant association for crown root number at 28 days on chromosome 6- 4.02 Mb. **(A)** Local Manhattan plot (top) and LD heat map (bottom) of QTL on chromosome 6, the blue solid line represents the candidate regions (243 kbp upstream and downstream of the peak SNP) and red dash line represents the candidate gene *OsGLPT2* (LOC_Os06g08170); **(B)** the synonymous and non-synonymous SNPs in the candidate gene *OsGLPT2* significantly associated with crown root number, and amino acid variations; and **(C)** variation in crown root number at 28 days indicated haplotypes of *OsGLPT2*. The phenotypic data are the average of two seasons. Significance level; *p < 0.05, **p < 0.01, ***p < 0.001, ****p < 0.0001. ns, non significant.

A QTL on chromosome 6, centered around 4.02 Mb was significantly associated with crown root number at 28 days (wet season). The pairwise LD correlations have highlighted a region from 3.71 Mb to 4.20 Mb as the length of this QTL (Figure 7). Taking into account the LD value of 243 kbp (Supplementary Table 7), the whole region was studied to identify the root growth genes located in this region. *OsGlpT2* (LOC_Os06g08170), a glycerol-3-phosphate transporter 2 gene was selected as the candidate gene for the haplotype study as there was a previous report of this gene’s role in Pi influx in plants (Xu et al., 2019). They are localized in the endoplasmic reticulum and plasma membrane acting as a transporter. Additionally, their spatiotemporal gene expression from the *RiceXpro* database suggested high levels of expression in the root at the vegetative stage. The gene harbored a total of 85 SNPs from the BAAP 2 million SNP database, of these, nine SNPs were in the exon region of the gene. In this exon region, non-synonymous SNPs were positioned in the following order; at 3,948,822 bp (C/T polymorphism), 3,950,213 bp (T/C polymorphism), 3,960,275 (G/A polymorphism), and 3,962,787 (T/A polymorphism) that resulted in the amino acid substitution from Arg (R) to Cys (C), Cys (C) to Arg (R), Gly (G) to Ser (S), and Ser (S) to Thr (T), respectively. The genotypes in this study were grouped into three haplotypes; hap A (*n* = 3), hap B (*n* = 153), and hap C (*n* = 43) for the above-mentioned SNPs. Even though the distinct superiority of haplotype A can be visualized, its reliability is rather less due to the lower number of representatives. However, hap B (12.31) and hap C (14.72) were significantly different than each other and the latter had a better proliferation of crown roots.

**FIGURE 8:**
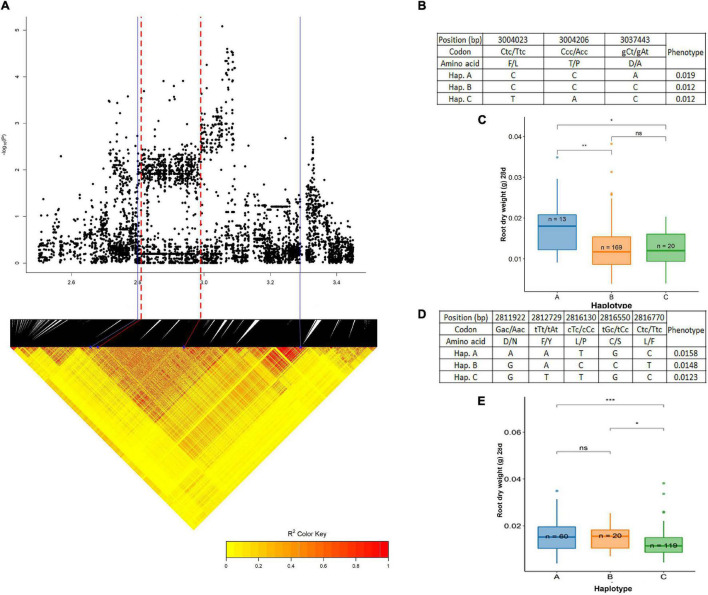
Significant association for root dry weight at 28 days on chromosome 3- 3.05 Mb. **(A)** Local Manhattan plot (top) and LD heat map (bottom) of QTL on chromosome 3, the blue solid line represents the candidate regions and red dash line represents the candidate genes LOC_Os03g05640 (*OsPht 1:2*) and LOC_Os03g06000 (*OsEXPA12*); **(B)** the synonymous and non-synonymous SNPs in the candidate gene *OsPht 1:2* significantly associated with root dry weight, and amino acid variations; **(C)** variation in root dry weight at 28 days indicated haplotypes of *OsPht 1:2*; **(D)** the synonymous and non-synonymous SNPs in the candidate gene *OsEXPA12* significantly associated with root dry weight, and amino acid variations; and **(E)** variation in root dry weight at 28 days indicated haplotypes of *OsEXPA12.* Significance level; *p < 0.05, **p < 0.01, ***p < 0.001, ****p < 0.0001. ns, non significant.

A QTL on chromosome 3, centered around the 3.05 Mb region was significantly associated with root dry weight at 28 days (wet season). The pairwise LD correlations (Figure 8A) have highlighted a region from 2.80 to 3.29 Mb to be the length of this QTL. An extensive search in this stretch of the region (Supplementary Table 8) was done to identify genes related to the root dry weight and was found to be a hotspot for expansin genes; LOC_Os03g06000 (*OsEXPA12*), LOC_Os03g06010 (*OsEXPA25*), LOC_Os03g06020 (*OsEXPA15*), LOC_Os03g06040 (*OsEXPA18*), and LOC_Os03g06060 (*OsEXPA20*). These genes have been reported to be root-specific and help in the development of outer cell layers (Lee and Kende, 2002). There was a total of 225 SNPs highlighted from this region in the BAAP 2 million SNP database, of which only 10 were in the exon region. There were three non-synonymous SNPs; 3,004,023 bp (C/T polymorphism), 3,004,206 bp (C/A polymorphism) in LOC_Os03g06010, and 3,037,443 bp (C/A polymorphism) in LOC_Os03g06060 that resulted in amino acids changes such as Lue (L) to Phe (F), Pro (P) to Thr (T), and Ala (A) to Asp (D), respectively. The cultivars used in this study were grouped into three haplotypes; hap A (*n* = 13), hap B (*n* = 169), and hap C (*n* = 20) (Figure 8B). The hap A (0.0186 g) group was significantly different and superior to hap B (0.0125 g) and hap C (0.0121 g) (Figure 8C). Another important gene located in this stretch LOC_Os03g05640 (*OsPht 1:2*) was chosen as the candidate gene for the haplotype study. It is reported to be a phosphate transporter expressed at the highest levels in the roots during the vegetative stage (*RiceXpro* database). In total, 53 SNPs were present in this region recognized in the BAAP 2 million SNP database. Of these, 11 SNPs were in the exon region of which 5 SNPs were responsible for non-synonymous changes. These were localized at 2,811,922 bp (G/A polymorphism), 2,812,729 bp (T/A polymorphism), 2,816,130 bp (T/C polymorphism), 2,816,550 bp (G/C polymorphism), and 2,816,770 bp (C/T polymorphism). These resulted in the following amino acid changes: Asp (D) to Asn (N), Phe (F) to Tyr (Y), Leu (L) to Pro (P), Cys (C) to Ser (S), and Leu (L) to Phe (F). Besides these, six synonymous mutations were occurring in this region. The studied cultivars formed three haplotypes based on the above-mentioned SNP; hap A (*n* = 60), hap B (*n* = 20), and hap C (*n* = 119) (Figure 8D). The haplotype C (0.0123 g) was inferior and significantly different than hap A (0.0156 g) and hap B (0.0148 g) while there was no significant difference between hap A and hap B (Figure 8E).

**FIGURE 9:**
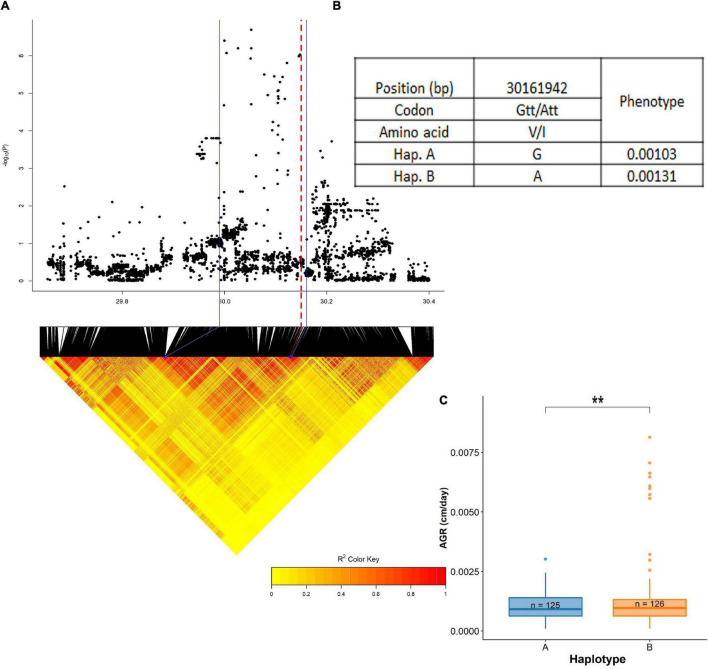
Significant association for absolute root growth on chromosome 4- 29.99–30.16 Mb. **(A)** Local Manhattan plot (top) and LD heat map (bottom) of QTL on chromosome 4, the blue solid line represents the candidate regions and red dash line represents the candidate gene LOC_Os04g50950 (*PTR6*); **(B)** the synonymous and non-synonymous SNPs in the candidate genes *PTR5* and *PTR6* significantly associated with absolute root growth, and amino acid variations; and **(C)** variation in absolute root growth indicated haplotypes of *PTR5* and *PTR6*. Significance level; *p < 0.05, **p < 0.01, ***p < 0.001, ****p < 0.0001. ns, non significant.

Another QTL on chromosome 4, stretched across a region from 29.99 to 30.16 Mb was significantly associated with absolute root growth (dry season) (Figure 9 and Supplementary Table 9). This region harbored two genes influencing root growth: LOC_Os04g50940 (*PTR5*) and LOC_Os04g50950 (*PTR6*). Both of these genes are reported to be contributing to plant growth by increasing nitrogen uptake (Fan et al., 2014; Huang et al., 2019), and also a high expression of these genes at the vegetative stage in roots has been reported (*RiceXpro* database). A total of 37 SNPs have been identified in the BAAP 2 million SNP database from this QTL region of which only three were located in the exon region. One of them was a non-synonymous change in SNP; 30,161,942 bp (LOC_Os04g50950) with A/G polymorphism leading to a change in amino acid; Ile (I) to Val (V). There were two synonymous SNPs located in this region. The cultivars were grouped into two haplotypes, hap A (*n* = 125) and hap B (*n* = 126). Haplotype A (0.00103) was superior to haplotype B (0.00131).

**FIGURE 10:**
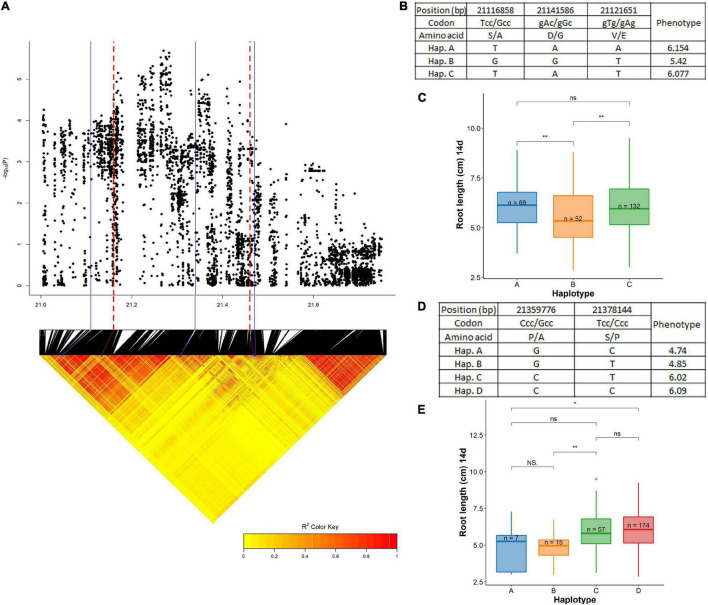
Significant association for root length at 14 days on chromosome 7- 21.27 Mb. **(A)** Local Manhattan plot (top) and LD heat map (bottom) of QTL on chromosome 7, the blue solid line represents the candidate regions and red dash line represents the candidate genes *OsRLCK234* (LOC_Os07g35280) and *UCL22* (LOC_Os07g35860); **(B)** the synonymous and non-synonymous SNPs in the candidate gene *OsRLCK234* significantly associated with root length, and amino acid variations; **(C)** variation in root length at 14 days indicated haplotypes of *OsRLCK234*; **(D)** the synonymous and non-synonymous SNPs in the candidate gene *UCL22* significantly associated with root length at 14 days, and amino acid variations; and **(E)** variation in root length at 14 days indicated haplotypes of *UCL22.* Significance level; *p < 0.05, **p < 0.01, ***p < 0.001, ****p < 0.0001. ns, non significant.

A QTL associated with root length at 14 days (dry season) was identified on chromosome 7 centered around 21.27 Mb having two LD blocks (21.12–21.30 and 21.35–21.46 Mb) (Figure 10 and Supplementary Table 10). The first LD block harbored *RLCK* family genes that had significant roles in influencing root traits under DSR. The major ones were *OsRLCK234* (LOC_Os07g35280) and *OsRLCK235* (LOC_Os07g35390). All of these genes have been reported to be having high levels of expression at the vegetative stage in the roots in the *RiceXpro* database. As this was a large segment of the chromosome, only the significant SNPs were considered for haplotype mapping. This included the following non-synonymous SNPs in the exon region: 21,116,858 bp (T/G polymorphism) in LOC_Os07g35280, 21,141,586 bp (A/G polymorphism) in LOC_Os07g35330, and 21,121,651 bp (T/A polymorphism) in LOC_Os07g35920 leading to changes in amino acids; Ser (S) to Ala (A), Asp (D) to Gly (G), and Val (V) to Glu (E), respectively. These SNPs grouped the studied haplotypes into three haplotypes; Hap A (*n* = 69), Hap B (*n* = 52), and Hap C (*n* = 132). The haplotype A (6.15 cm) was superior and significantly different than the lowest mean Haplotype B (5.42 cm) and C (6.07 cm). Another candidate gene associated with root length (at 14 days) was identified in the next LD block at 21.46 Mb. This region harbored LOC_Os07g35860 (*UCL22*) associated significantly with root length at 14 days. The significant SNPs were identified for the haplotype study out of which two were non-synonymous in the exon region located at 21,359,776 bp (C/G polymorphism) and 21,378,144 bp (T/C polymorphism) that led to changes in amino acids such as Pro (P) to Ala (A) and Ser (S) to Pro (P), respectively. These SNPs have grouped the cultivars in this study into the four haplotypes; Hap A (*n* = 7), Hap B (*n* = 15), Hap C (*n* = 57), and Hap D (*n* = 174). Hap D (6.09 cm) was the superior group with a high mean significantly different than the low mean group; Hap A (4.74 cm).

## Discussion

The aerobic conditioned DSR system is regarded as a resource-efficient and eco-friendly system. The soil environment and root dynamics are key players in this system. In the aerobic system, the soil is usually in the oxidized state apart from the occasionally reduced condition in the wet season (due to precipitation). This difference not only alters the root extensions but also the transporters operating at the cellular level. In such a soil environment, it is crucial to realize the importance of both genes and the transporters involved in root development. At the nutrient level, this study highlights the importance of nitrogen in the soil maintained under deficient conditions. For instance, transporters like *AMT1*; an ammonium transporter (Sonoda et al., 2003) is active in the reduced soil whereas *NRT/PTRs*; a nitrate transporter family (Fan et al., 2017) is functional in the case of the oxidized soil. Both these related transporters are significantly associated with their characteristics seasons in our study; nitrate transporters like *NRT2.1* (LOC_Os02g02170), *NRT2.2* (LOC_Os02g02190), *NPF6.5* (LOC_Os10g40600), *NPF7.4* (LOC_Os04g50940), and *PTR6* (LOC_Os04g50950) were expressed in the dry season whereas transporters like *AMT1.2* (LOC_Os02g40730), *AMT1.3* (LOC_Os02g40710), and *AMT3.2* (LOC_Os03g62200) were significant in the wet season. Another important aspect of the experiment was the field maintained under nitrogen-deficient conditions. In the DSR system, the weed menace is a major problem, and to address this situation, there have been suggestions to avoid the basal dose of nitrogen. Instead of the basal dose, it is recommended to apply nitrogen at a later stage (preferably at 14 DAS) which gives the crop a head start against the weeds (Thind et al., 2018). Nitrogen, when applied as a basal dose in the DSR system, leads to a loss of the nutrient as the plant is unable to uptake it and serves as a booster dose for the weeds. Delaying this basal dose is found to significantly improve the N uptake and yield (Qi et al., 2012) because when applied as basal, the immature root system of the rice seedling at the early stage (prior to 14 days) is unable to completely utilize the N-fertilizer (Wang et al., 2017). It is also reported that the basal application can cause ammonia toxicity resulting in a reduction in early-seedling vigor (Haden et al., 2011), an important trait to tackle the weeds, under DSR. The foremost requirement at the early stages is quick root proliferation which can be facilitated by phosphorus uptake through various genes and transporters (Bates and Lynch, 2001). The results of previous works have also highlighted the importance of P regulating genes that helped in the initial root development. This was visualized in this study with the comparatively higher association of P transporters in this GWAS for root vigor like *OsPHO1;2* (LOC_Os02g56510), *OsPht1;*1 (LOC_Os03g05620), *GLPT2* (LOC_Os06g08170), and *OsMYB30* (LOC_Os02g41510) among many others. We can put forward the idea that these genes and transporters may play a pivotal role in the initial root development and nutrient uptake even in nitrogen-deficient soil. The BAAP population under study displayed a wide range of variability for all the root morphological characters and a clear significant difference between the two different soil conditions in the dry and wet seasons. The dry season had high mean values for all the root traits that include root number, length, and dry weight at both 14 and 28 days. This reflects the need of the plant for better root proliferation under moisture stress in the dry season as compared to the wet season. A locus LOC_Os02g50350 encoding the gene *OsDHODH1* was found to be significantly associated with root vigor trait in the GWAS study specifically in the dry season. The overexpression of *OsDHODH1* is related to drought stress response in rice and could serve as a potential candidate gene for drought tolerance in rice (Rolly et al., 2019), especially in DSR conditions. Another locus that was associated with similar conditions was also found to be expressed and associated with root number at 14 days in the dry season; LOC_Os05g03760 coding for C3H33 (Pandey and Kim, 2012). Other drought stress response loci that had significant associations with various root traits were LOC_Os04g50060 (*OsGRAS23*) (Xu et al., 2015), LOC_Os01g45990 (*OsAKT1*) (also involved in potassium uptake) (Ahmad et al., 2016), LOC_Os08g44350 (*OsAHP1*) (Sun et al., 2014), LOC_Os07g26660 (*OsPIP2;5*) (Deng et al., 2020) (Lian et al., 2006), LOC_Os05g14240 (*OsTIP4;1*) (Li et al., 2008) in the dry season and LOC_Os03g60080 (*OsNAC1*) (You et al., 2014), LOC_Os02g32140 (*OsERF107*), and LOC_Os04g46220 (*OsERF1*) (Hu et al., 2008) in the wet season. These genes can be further studied to identify their interaction with other yield traits and exploited for enhancing drought tolerance in aerobic DSR conditions.

The nutrient uptake in the initial phase of growth is mostly from the top or shallow layers of the soil, of the immobile nutrients such as P, Zn, Fe, etc. along with a minor portion of N. Many loci have been significantly associated with various nutrient acquisition processes associated with a number of root traits in the dry and wet seasons. Some major ones were LOC_Os08g10630 (*ZIP4*) (Ishimaru et al., 2005), LOC_Os05g10940 (*ZIP7*) (Yang X. et al., 2009), LOC_Os11g04030 (*YSL2*) (Kobayashi et al., 2014), LOC_Os02g43410 (*YSL15*) (Kobayashi et al., 2014), LOC_Os04g58760 (*CASP1*) (Wang et al., 2019), LOC_Os11g04020 (*TOM1*) (Kobayashi and Nishizawa, 2012), and LOC_Os11g04030 (*TOM2*) (Nozoye et al., 2015) that enhanced iron and zinc uptake, LOC_Os02g41510 (OsMYB30) (Yang et al., 2018), LOC_Os02g56510 (*OsPHO1;2*) (Secco et al., 2010), LOC_Os06g08170 (*GLPT2*) (Xu et al., 2019), LOC_Os03g05640 (*OsPT2*) (Zhang et al., 2014), LOC_Os06g21920 (*OsPht1;9*) LOC_Os06g21950 (*OsPht1;10*), (Wang et al., 2014), and LOC_Os12g38770 (*PAP1B*) (Lea et al., 2007) in relation to phosphorus uptake, LOC_Os03g37840 (*OsHAK16*) (Chen et al., 2015), LOC_Os06g15910 (OsHAK24) (Yang Z. et al., 2009), LOC_Os09g38960 (*OsHAK18*) (Wang et al., 2020), LOC_Os02g02170 (*NRT2.1*) (Chen et al., 2019), LOC_Os02g02190 (*NRT2.2*) (Yan et al., 2011), LOC_Os03g59570 (*OsIPT4*), LOC_Os04g50940 (*PTR5*), LOC_Os04g50950 (*PTR6*), LOC_Os12g43440 (*TOND1*) (Zhang et al., 2015), LOC_Os02g40710 (*OsAMT1.3*), LOC_Os02g40730 (*OsAMT1.2*) (Kumar et al., 2003), and LOC_Os03g62200 (*OsAMT3.2*) (Su-Mei et al., 2012) for nitrogen uptake. Apart from the above, nutrient acquisition was also aided by the mycorrhizal genes expressed during both seasons. LOC_Os02g03150 (AM3), LOC_Os02g03190 (AM31), and LOC_Os02g03410 (AM24) (Gutjahr et al., 2008) in the dry season and LOC_Os03g10620 (DI4) (Takeuchi et al., 2018), LOC_Os07g22710 (OsCPK18) (Campos-Soriano et al., 2011), and LOC_Os10g18510 (AM34) (Gutjahr et al., 2008) in wet season. The aerobic conditions maintained in this study must have supported the growth of soil microbes including mycorrhiza *via* root exudates (Iqbal et al., 2021). This can be considered as one of the reasons for the difference in root dry weight in the anaerobic wet season where the mycorrhizal activity is delimited and the aerobic dry condition in the dry season.

The crown root number, both at 14 and 28 days, was higher in the dry season with a significantly high genotypic contribution (29.27–38.72%). There was a significant interaction between the genotypes and seasons for the crown root number at 28 days. Some of the important QTL regions that harbored genes controlling the development of crown root were found in association with root traits in this study, few important ones were LOC_Os01g58420 (*ERF3*) (Zhao et al., 2015), LOC_Os02g32140 (*ERF107*) (Rashid et al., 2012), LOC_Os02g35180 (*OsRR2*) (Zhao et al., 2009), LOC_Os03g06620 (*OsARD2*) (Yang et al., 2004), LOC_Os07g03250 (*OsCRL5*) (Kitomi et al., 2011), and LOC_Os07g31450 (*OsCRL6*) (Wang et al., 2016). This highlights the fact that the rice plant would require a higher number of shallow roots against a few deep roots for the initial stages of nutrient uptake (especially the soil immobile nutrients).

There was a clear difference between the two seasons and the studied root traits as evident from the PCA, where they formed separate localized clusters indicating their differences with respect to root vigor-related traits. In the wet season, the top layer of soil readily offers moisture that aids in the nutrient acquisition required at the initial stages of growth. But in the dry season, due to the lack of precipitation, the plant must have prioritized root growth to maintain the moisture, and nutrient acquisition to accumulate shoot biomass without any compromise under the aerobic growth conditions. This fact is supported by the observed shoot dry weight mean maintained at 0.63 g at 28 days during both seasons. Interestingly, the high correlation values between the root dry weight and shoot dry weight at 28 days also point out the importance of a robust root system to keep up with the shoot biomass accumulation. Many root growth influencing QTL regions were significantly associated with different traits in this study, some of the major genes present in these regions were LOC_Os03g06000 (*OsEXPA12*) (Lee and Kende, 2002), LOC_Os07g35280 (*OsRLCK234*), LOC_Os07g35390 (*OsRLCK235*) (Vij et al., 2008), and LOC_Os01g15340 (*OsRAA1*) (Han et al., 2008). Cultivars expressing such genes grow their roots vigorously to maintain shoot growth and can be of great value for developing ideal cultivars for direct-seeded conditions.

The main aim of the study was to visualize the phenotypic variations of the root and then associate them with the variations at the SNP level, candidate genes regulating them, study their functional annotations, and the diverse haplotypes that they form in the BAAP population. Many such QTLs controlling root vigor are already identified pertaining to seedling establishment, root growth, and nutrient uptake. In this study, we found a total of 97 QTL regions strongly associated with different root traits controlling, nutrient, hormonal and growth dynamics. Few of the QTL regions were expressed repeatedly; the QTL region 3.00–3.09 Mb of chromosome 3 was associated with root dry weight 28 days that possessed two candidate genic regions (expansin genes and phosphate transporters). Few of these were already reported while few other regions identified in this study have not been previously reported, for instance, the two haplotype regions (which can be considered as two LD blocks) associated with root length at 14 days from the region of 21.11 to 21.37 Mb on chromosome 7.

### Haplotypes and Candidate Genes

QTL regions and the candidate genomic regions were selected on the level of association, LD plots, and Manhattan peaks. The selection criteria for these genes were organ-specific, i.e., governing the growth and proliferation of the roots. Six different genomic regions are reported in detail in this study, of which three were related to root dry weight, one for crown root number, two for root length, and one for AGR. Two haplotypes concerning root dry weight were identified on chromosome 3, within the QTL region spanning from 3.00 to 3.09 Mb. The proposed candidate genes for these two haplotypes were of the family *Pht* and *EXPA*. The haplotype for phosphate transporters included LOC_Os03g05620 (*OsPhT1*) and LOC_Os03g05640 (*OsPhT2*). Among these genes, *OsPhT1*, a high-affinity phosphate transporter is constitutively expressed and involved in the transport of Pi across plasma membrane at the soil root interface while *OsPhT2*, a low-affinity phosphate transporter involved in Pi transport between subcellular components is induced due to Pi deficiency in the roots (Mahender et al., 2018). The non-synonymous SNP changes in the exon region of these regions grouped the BAAP cultivars into three different haplotypes. The haplotype C was mutant (compared to *Nipponbare*) at all the five SNP positions with the lowest mean root dry weight present in the highest number of *aus* cultivars. The haplotype A was significantly different having the highest mean and the mutant SNPs at three positions were for LOC_Os03g05640 (*OsPhT2*), these changes were from Leucine to Proline, Cysteine to Serine, and leucine to phenyl alanine. However, these three changes were similar to that of Haplotype C, but the difference existed in the initial two SNPs representing the locus LOC_Os03g05630 (expressed protein). The substitution here was Aspartic Acid to Asparagine and Phenylalanine to Tyrosine at the positions 2,813,584 and 2,811,999 bp, respectively. Thus, the loci need to be further studied for their putative role in root growth. The QTL region strongly associated with root dry weight harboring *expansin* genes was overlapping with the above-discussed region. This region represented the candidate genes such as LOC_Os03g06000 (*EXPA12*), LOC_Os03g06010 (*EXPA25*), LOC_Os03g06020 (*EXPA15*), LOC_Os03g06040 (*EXPA18*), and LOC_Os03g06060 (*EXPA20*). The *expansin* proteins help in root cell wall expansion by loosening the cell wall and expansion of epidermal cells to form long coleorhiza hairs. Of the three non-synonymous substitutions in this region, two are in the *EXPA25* exon region and one is in the *EXPA20* exon region. These three SNPs have grouped the *aus* cultivars into three different haplotypes. The most superior group with high mean root dry weight is Haplotype A with mutations in the SNP positions; 3,004,023 and 3,004,206 bp, one in each of the two genes mentioned above while the third position 3,037,443 bp resembled the wild-type. This led to a change in amino acids from Leucine to Phenylalanine in both positions. The gene *EXPA20* is highly expressed in the roots, especially in the early vegetative stages as suggested by the transcriptome analysis using microarray in the *RiceXPro* database. Crown root number at 28 days had a strong association with a QTL on chromosome 6 with a high peak centered around the region 4.02 Mb. LOC_Os06g08170 (*GLPT2*) was chosen as the candidate gene coding for a Glycerol-3-Phosphate transporter involved in the Pi release from the vacuoles to the cytoplasm and helps to adapt to low-Pi stress, this, in turn, modulates the root development. A receptor-like-protein kinase and a protein phosphatase were also present in this region within the QTL. Of the four non-synonymous SNPs, one (3,962,787 bp) was in the GLPT2 exon region, two (3,948,822 and 3,950,213 bp) in the protein phosphatase gene, and one (3,960,278 bp) in the receptor-like-protein kinase locus, leading to amino acid substitutions; Arginine to Cysteine, Cysteine to Arginine, Glycine to Serine, and Serine to Threonine, respectively. These changes grouped the *aus* cultivars into three haplotypes, the superior one haplotype A was the rare group with a high number of crown roots and significantly different from the others. AGR, one of the relative traits had a strong association with a QTL region on chromosome 4, this region had two important peptide transporters adjacent to each other; LOC_Os04g50940 (*PTR5*) and LOC_Os04g50950 (*PTR6*). These two genes are reported to be nitrogen transporters with high expression in the root during the vegetative stages. However, there was only one non-synonymous SNP in this region changing the amino acid from the wild-type isoleucine to mutant type valine in the *PTR6* exon sequence.

Two separate LD blocks were identified adjacent to each other regulating root length of 2 weeks old around the region from 21.12 to 21.46 Mb on chromosome 7. These two regions harbored gene loci that were constantly upregulated and had a strong association with root length, however, there were no earlier reports regarding these. The first block had the loci LOC_Os07g35280 (*OsRLCK234*) and LOC_Os07g35390 (*OsRLCK235*) and according to the microarray transcriptomic data available in RiceXpro, both of them are highly expressed in the vegetative roots. We have found three non-synonymous SNPs in this entire stretch; 21,116,858, 21,121,651, and 21,141,586 bp leading to amino acid changes; Alanine to Serine, Glutamic acid to Valine, and Glycine to Aspartic acid, respectively. These substitutions divided the cultivars studied here into three haplotypes. The superior haplotype A had a mutation at 21,141,586 bp (Glycine to Aspartic acid) in the exon region of LOC_Os07g35330, which is identified as DUF26 kinases having homology with DUF26 containing loci. The other LD block around the same region associated with root length also had many loci without proper functional annotation that are yet to be validated. One of the major genes in this region was the LOC_Os07g35860 (UCL22). Only two non-synonymous SNPs were identified located at 21,359,776 and 21,378,144 bp leading to a change in amino acids from Alanine to Proline and Alanine to Serine, respectively. With the two non-synonymous substitutions, the *aus* cultivars in this study were grouped into four haplotypes. The superior haplotype was haplotype D, with non-synonymous change at 21,378,144 bp located in the exon regions of LOC_Os07g35690. As they displayed high peaks in the Manhattan plots and had significant LD values, we speculate this region to be coding for some major root length governing gene and can serve as a candidate region for developing vigorous root growing varieties for DSR condition.

### Identifying Superior Donors for a Haplotype-Based Breeding Program

The identification of superior haplotypes also helped us to highlight well-performing genotypes from the BAAP panel that could serve as donors for various early root vigor traits which can be exploited in a haplotype-based breeding program to design ideal plants with ideal early root vigor. The root dry weight or the biomass accumulated over 28 days, root length, crown root number, and AGR root were the major traits that were analyzed for different genes in the haplotype studies, is an important criterion for categorizing ideal performing genotypes. The genotypes Lara (BAAP251), AUS 68 (BAAP29), and AUS 294 (BAAP60) were considered superior based on their representation in the superior haplotypes of the studied traits. The root lengths of these genotypes were 7.12, 6.94, and 6.29 cm at 14 days which was higher than the superior group average. The root dry weight at 28 days of Lara was 0.0168 g with AGR of the root being 0.0013 g day^–1^, while the root dry weight of AUS 68 and AUS 294 was 0.0208 and 0.0121 g, respectively. The AGR of root painted a different picture as it was high for Lara (0.0013 g day^–1^) but low in the case of AUS 68 (0.0008 g day^–1^) but with high root biomass. Such a trend further sheds light on the fact that biomass accumulation in the initial period must be very high, i.e., in the first 14 days. Such a trait is highly essential for a plant to establish itself successfully under the direct-seeded condition of rice. Two lines represented across inferior groups were also identified, the allelic mutations of these genotypes could serve as markers for a casual phenotype. These were Polman and NP97 with root dry weights of 0.0045 and 0.0054 g, respectively. The average number of crown roots was 7.2 and 5.4 for Polman and NP97, respectively.

### Proposing Genes Involved in Root Architecture and Nutrient Acquisition for Early Root Vigor Under Direct Seeded Rice Condition

**FIGURE 11:**
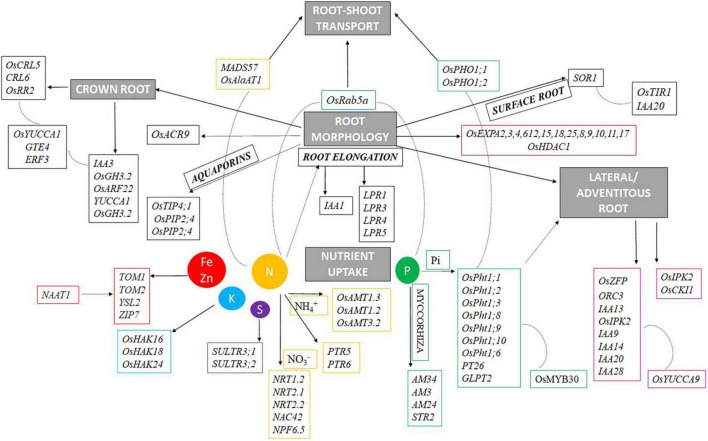
Proposed regulatory network mechanism involved in root architecture and nutrient acquisition for early root vigor in rice under DSR conditions.

**FIGURE 12:**
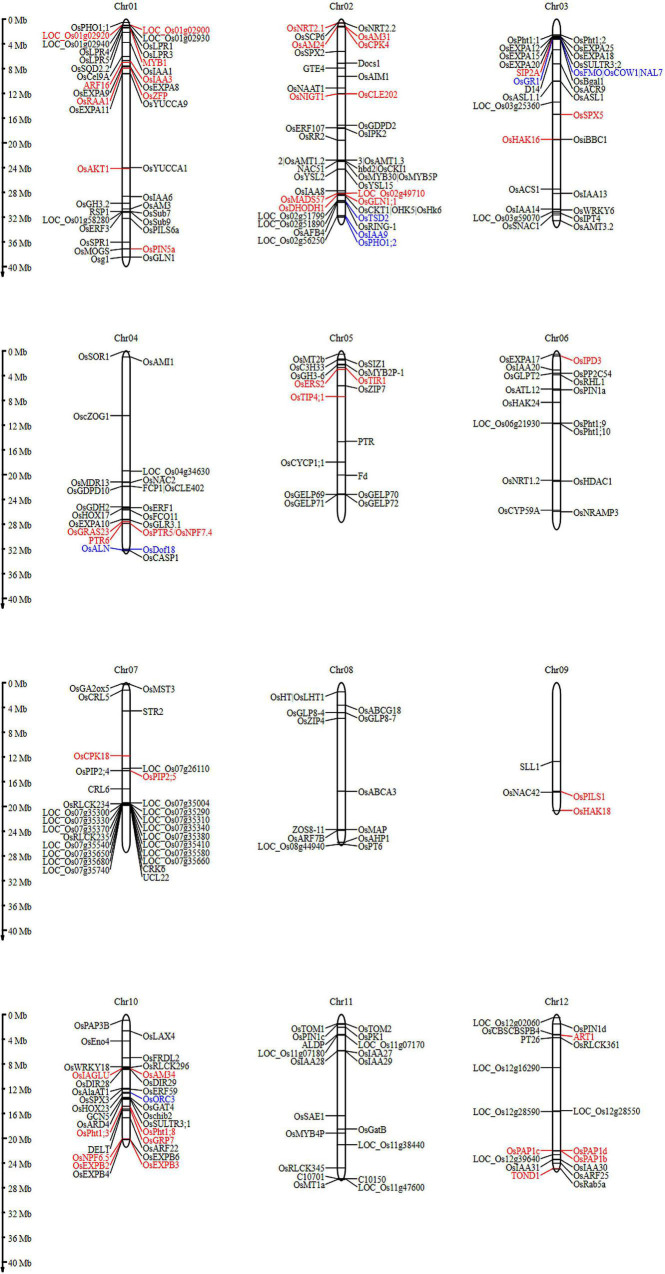
Root architecture and nutrient acquisition genes involved in young rice seedlings under DSR conditions detected in this study were presented in 12 chromosomes. Candidate genes found in multiple traits and across seasons were indicated by red and blue fonts, respectively.

Early root vigor is defined by a few major characteristic features of the root such as root proliferation (development of crown roots, seminal roots, lateral roots, root hairs, etc.), nutrient acquisition by the roots, and its transport to plant parts above the ground. Thus, to model a plant with ideal early root vigor traits, one needs to understand the key players involved in regulating the above-mentioned features of the root. A list of 275 unique putative genes related to root architecture and nutrient acquisition were identified in this study from 97 QTLs. The identified genes associated significantly with various traits in this study have been studied to understand their role in the manifestation of various root characteristics (Figure 11) and are represented by chromosome wise (Figure 12). The most important role of the root in the early period or the first 2 weeks is to establish itself and facilitate nutrient uptake in the dry direct-seeded condition. P transporter activity starts as early as 3 days after germination (Julia et al., 2018), this not only facilitates P uptake but also acts as a root growth enhancer. The P uptake is facilitated by Pi transporters such as *OsPht1;1, OsPht1;2, OsPht1;3, OsPht1;8, OsPht1;9, OsPht1;10, OsPht1;6, PT26*, and *GLPT2*. The *Pht* transporters (both high- and low-affinity transporters) have been known to play a wide range of roles in Pi uptake and its translocation to the shoots through *OsPHO1:1* and *OsPHO1:2*. A slight variation in the protein sequence of the *PHT1* proteins can lead to varying degrees of Pi acquisition (Liu et al., 2008). In this process, the phosphate transporters are regulated by the transcription factor *OsMYB30* (Yang et al., 2018), and involved in root-system architecture. Additionally, mycorrhizal genes, *AM3, AM24, AM34*, and *STR2* were also expressed in the study which again is involved in the Pi uptake in plants. LPR genes (*LPR1, LPR3, LPR4*, and *LPR5*) (Svistoonoff et al., 2007; Cao et al., 2016), multicopper oxidase domain-containing protein, control root length in response to the phosphorus starvation. Similarly, other nutrient uptake genes were *SULTR3:1* and *SULTR3:2* for Sulfur, *OsHAK16, OsHAK18*, and *OsHAK24* genes for potassium transport) involved under upland aerobic conditions (Wang et al., 2019) and TOM1, TOM2, YSL2, and ZIP7 for Iron and Zinc transport. NAAT1 gene, strongly upregulated by iron deficiency helps in the synthesis of deoxymugineic acid synthase (DMS) that helps in the uptake of iron (Cheng et al., 2007). Nitrogen uptake has different dynamics and regulation activity depending on the soil environment. In the case of the dry season, nitrate transporter plays a major role such as *NRT1.2*, *NRT2.1*, *NRT2.2*, *NAC42*, and *NPF6.5*. But during the wet season, ammonium transporters, such as *OsAMT1.3*, *OsAMT1.2*, and *OsAMT3.2*, play a lead role in nitrogen acquisition activity. During this scenario, the plant also faces intermittent moisture stress, aquaporin genes like *OsTIP4;1*, *OsPIP2;4*, and *OsPIP2;4*. The nutrient and moisture uptaken by the roots must be translocated to the shoots, this activity is sometimes facilitated by the transporter molecule itself or sometimes by unique genes. In this study, we found the expression of *MADS57, OsAlaAT1*, and *OsRab5a* involved in nitrogen translocation while *OsPHO1;1* and *OsPHO1;2* are involved in the Pi translocation to the shoots. All the above-mentioned functions and gene activities can only be facilitated with an ideal root system. Thus, the genes that dictate the temporal and structural proliferation of the root and its appendages such as seminal root, crown root, lateral roots, root hairs, etc., are of prime importance. The roots that develop early in the seedling stages helping in the seedling establishment and nutrient uptake are the crown roots. The nutrients present in the shallow layers of the soil are mostly acquired during the seedling stages. The growth and development of these crown roots are controlled by the expression of genes like *OsCRL5, OsCRL6, OsRR2, IAA3, OsGH3.2, OsARF22*, and *YUCCA1*. These crown roots controlling genes are in turn regulated by *OsYUCCA1, GTE4*, and *ERF3*. Surface roots are also essential in this context to mine out nutrients from the top layers of the soil. Genes like *SOR1, TIR1*, and *IAA20* help in surface rooting by altering auxin activity and the gravitropic response of the roots. Another gene OsACR9 protein was distributed in the epidermis, exodermis, sclerenchyma, and vascular parenchyma cells of the root, developing the root morphology. Similarly, *OsEXPA2, 3, 4, 6, 12, 15, 18, 25, 8, 9, 10, 11, 17*, and *OsHDAC1* help in root cells and seedling root growth, respectively. Lateral roots increase the reach of the roots in the soil, such genes include *OsZFP*, *ORC3 IAA13, OsIPK2, IAA9, IAA14*, and *OsCK11*. These genes for lateral roots are in turn governed by *OsYUCCA9*.

## Conclusion

In this study, the *aus* population was evaluated for early root vigor under direct-seeded conditions over two seasons (dry and wet seasons). The studied root traits exhibited significantly higher mean values in the dry season as compared to the plants grown during the wet season. The root dry weight at 28 days had a positive association with RGR, AGR, and CGR. Notably, shoot dry weight at 28 days had a high association with root dry weight at 28 days. The GWA study of 12 root traits and six growth rate parameters identified 275 unique putative genes expressed in roots related to root architecture and nutrient acquisition under direct-seeded conditions from 97 QTLs. Among them notably, ammonium transporters played a lead role in the nitrogen acquisition activity under high precipitation, while nitrate transporters were found expressed during the dry season. Compared to N-related genes, a maximum number of P-related genes, transporters, and transcription factors were found to play a major role in early root vigor under direct-seeded conditions. This suggests that P-related genes may have a significant role in root proliferation at an early stage of rice seedlings. The haplotype analysis serves to identify superior haplotypes for notable genes and novel QTLs involved in early root vigor. Besides, the donors with superior haplotypes that were identified would be useful for future studies to design ideal plants with ideal early root vigor for direct-seeded conditions to aid haplotype-assisted breeding.

## Data Availability Statement

The original contributions presented in this study are included in the article/Supplementary Material, further inquiries can be directed to the corresponding author.

## Author Contributions

AA designed and conducted the field experiments. AT developed the GWAS pipeline. SP, AA, SS, AP, and GN performed the data analysis and wrote the first draft of the manuscript. All authors read and approved the manuscript.

## Conflict of Interest

The authors declare that the research was conducted in the absence of any commercial or financial relationships that could be construed as a potential conflict of interest.

## Publisher’s Note

All claims expressed in this article are solely those of the authors and do not necessarily represent those of their affiliated organizations, or those of the publisher, the editors and the reviewers. Any product that may be evaluated in this article, or claim that may be made by its manufacturer, is not guaranteed or endorsed by the publisher.

## Footnotes

1
https://www.fao.org/worldfoodsituation

2
https://snp-seek.irri.org/

3
http://rice.uga.edu/pub/data/Eukaryotic_Projects/o_sativa/annotation_dbs/pseudomolecules/version_7.0/

## Supplementary Material

Supplementary Figure 1 Manhattan plots from GWA mapping of early root traits at 14 and 28 days under DSR condition of BAAP population in wet season. Benjamini–Hochberg adjusted probabilities > 0.1 are highlighted in the red dot. The diagonal blue line shown on QQ Plots represents a 1:1 agreement between expected probability. Candidate gene of notable QTL is shown on the top.

Supplementary Figure 2 Manhattan plots from GWA mapping of early root traits at 14 and 28 days under DSR condition of BAAP population in dry season. Benjamini–Hochberg adjusted probabilities > 0.1 are highlighted in the red dot. The diagonal blue line shown on QQ Plots represents a 1:1 agreement between expected probability. Candidate gene of notable QTL is shown on the top.
