# Supplementary figures and images for: Superior Haplotypes for Early Root Vigor Traits in Rice Under Dry Direct Seeded Low Nitrogen Condition Through Genome Wide Association Mapping
Source: Front Plant Sci. 2022 Jul 8;13:911775. doi: 10.3389/fpls.2022.911775 (PMC9305665; doi:10.3389/fpls.2022.911775)

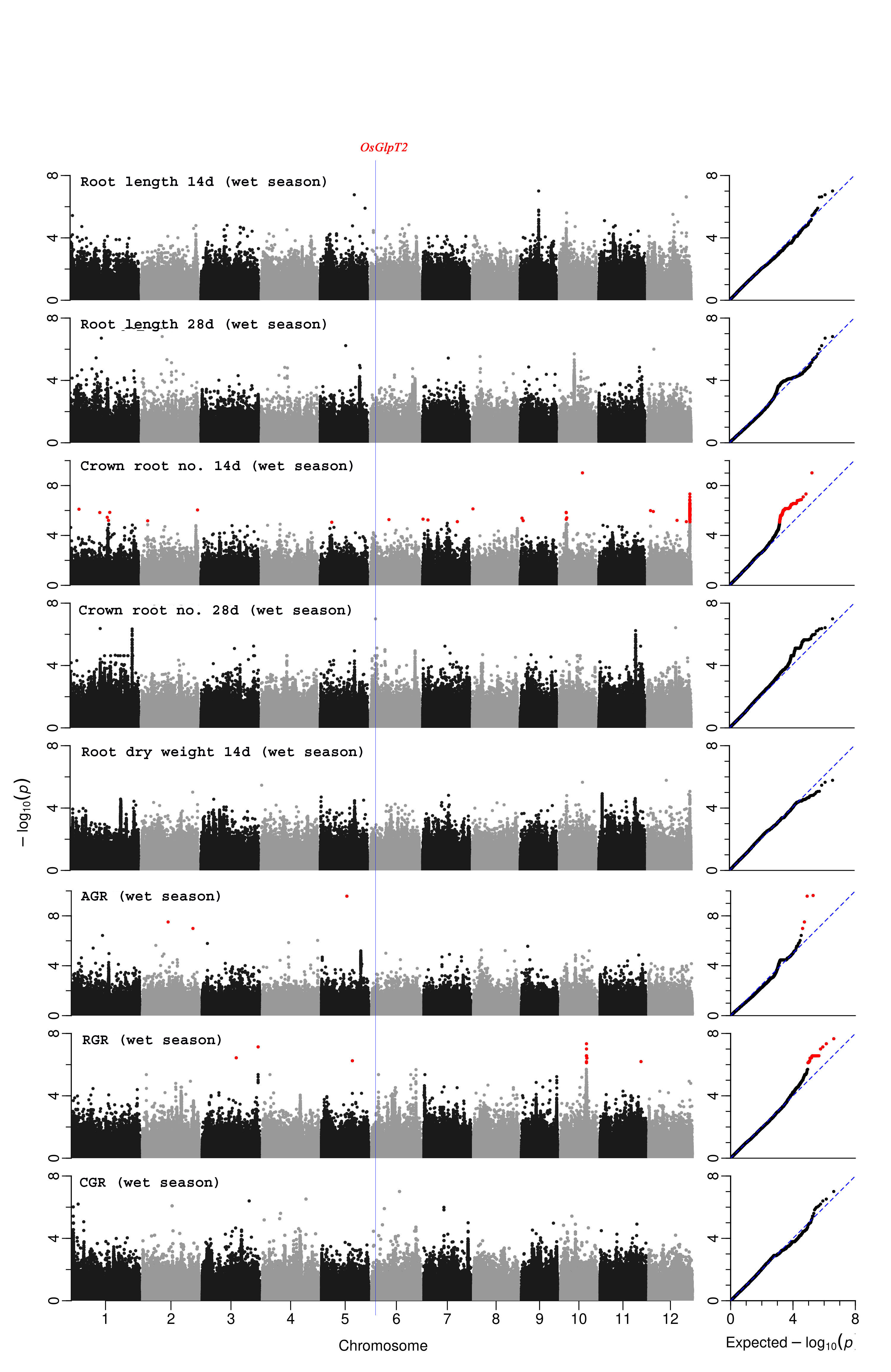

Supplement: Supplementary Figure 1 — Manhattan plots from GWA mapping of early root traits at 14 and 28 days under DSR condition of BAAP population in wet season. Benjamini–Hochberg adjusted probabilities > 0.1 are highlighted in the red dot. The diagonal blue line shown on QQ Plots represents a 1:1 agreement between expected probability. Candidate gene of notable QTL is shown on the top. [file Image_1.JPEG]

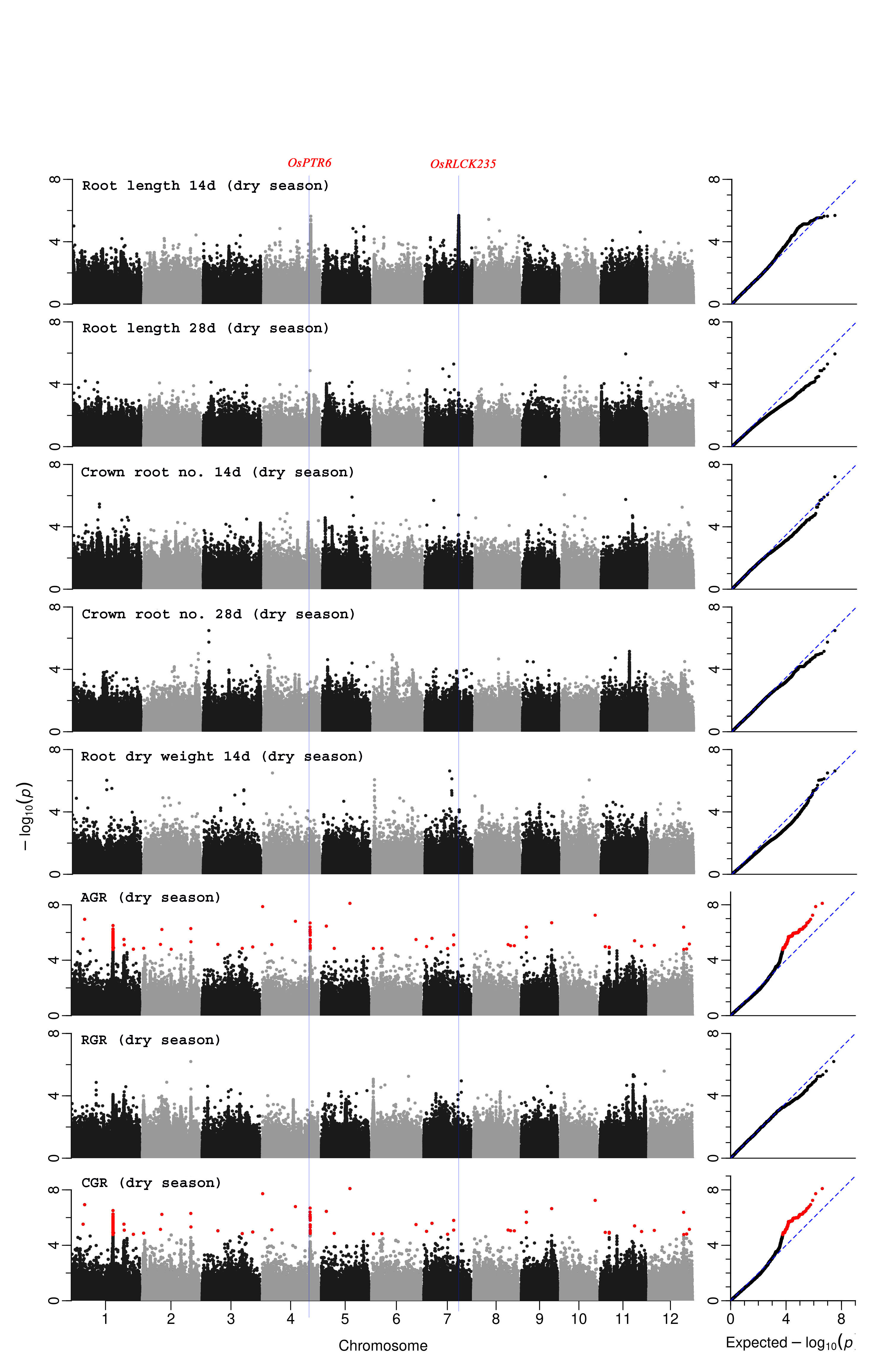

Supplement: Supplementary Figure 2 — Manhattan plots from GWA mapping of early root traits at 14 and 28 days under DSR condition of BAAP population in dry season. Benjamini–Hochberg adjusted probabilities > 0.1 are highlighted in the red dot. The diagonal blue line shown on QQ Plots represents a 1:1 agreement between expected probability. Candidate gene of notable QTL is shown on the top. [file Image_2.JPEG]
